# Comparison of Risk Scoring Systems to Predict the Outcome in ASA-PS V Patients Undergoing Surgery: A Retrospective Cohort Study

**DOI:** 10.1097/MD.0000000000003238

**Published:** 2016-04-01

**Authors:** Derya Arslan Yurtlu, Murat Aksun, Pınar Ayvat, Nagihan Karahan, Lale Koroglu, Gülcin Önder Aran

**Affiliations:** From the Anesthesiology and Reanimation Department, İzmir Atatürk Education and Research Hospital, İzmir, Turkey.

## Abstract

Operative decision in American Society of Anesthesiology Physical Status (ASA-PS) V patient is difficult as this group of patients expected to have high mortality rate. Another risk scoring system in this ASA-PS V subset of patients can aid to ease this decision.

Data of ASA-PS V classified patients between 2011 and 2013 years in a single hospital were analyzed in this study. Predicted mortality of these patients was determined with acute physiology and chronic health evaluations (APACHE) II, simplified acute physiology score (SAPS II), Charlson comorbidity index (CCI), Porthsmouth physiological and operative severity score for enumeration of mortality and morbidity (P-POSSUM), Surgical apgar score (SAS), and Goldman cardiac risk index (GCRI) scores. Observed and predicted mortality rates according to the risk indexes in these patients were compared at survivor and nonsurvivor group of patients. Risk stratification was made with receiver operator characteristic (ROC) curve analysis.

Data of 89 patients were included in the analyses. Predicted mortality rates generated by APACHE II and SAPS II scoring systems were significantly different between survivor and nonsurvivor group of patients. Risk stratification with ROC analysis revealed that area under curve was 0.784 and 0.681 for SAPS II and APACHE II scoring systems, respectively. Highest sensitivity (77.3) is reached with SAPS II score.

APACHE II and SAPS II are better predictive tools of mortality in ASA-PS V classified subset of patients. Discrimination power of SAPS II score is the best among the compared risk stratification scores. SAPS II can be suggested as an additional risk scoring system for ASA-PS V patients.

## INTRODUCTION

Together with the increasing elderly population in developed countries, more surgical interventional procedures are performed on patients who have more comorbid diseases, thus resulting with an increase in morbidity and mortality. The American Society of Anesthesiology Physical Status (ASA-PS) evaluation scale is the most widely used risk classification system in the preoperative evaluation of patients and it ensures the unity of data.^1^ The ASA-PS scale was revised, simplified, and used to evaluate perioperative mortality.^2,3^ Many studies have revealed the correlation between ASA-PS and perioperative mortality.^4,5^

ASA classification investigates the physical status of patients in 6 groups, with patients evaluated as ASA-PS V forming a patient group with expected mortality whether surgery occurs or not.^3^ Patients within ASA-PS V group undergoing surgery are expected to have high mortality rates. Especially in ASA-PS V group patients, making the decision for major surgery involves problems for the surgeon. In these patients, surgery is completed for treatment and largely for palliative aims to lengthen life.^2^

Although ASA-PS classification is simple and easy, interpretative differences by users in evaluating the patients’ physical status may cause subjectivity. As a result, in addition to ASA-PS classification, the search for risk scoring systems to strengthen operative mortality estimation continues.^2^

Our study is based on the idea that using an additional independent risk scoring system for ASA-PS V group patients also correlates with short term mortality. As a result, we researched 6 intensive care and surgical risk evaluation systems for ASA-PS V group patients to determine which was superior in predicting mortality. Thus, we aimed to find an appropriate risk scoring system supporting the evaluation of ASA-PS V classified patients.

## METHOD

After receiving local ethics committee’ permission (İzmir Katip Çelebi University Non-interventional Clinical Research Ethics Committee Chair: Prof. Dr. Recep Sütçü, Decision no/Date: 99/26.04.2013), the patient information from ASA-PS V patients who underwent operations at our hospital from 2011 to 2013 was retrospectively investigated from files and electronic database records. ASA-PS V classified patients were determined from files and electronic database. These ASA-PS V patients were investigated for age, sex, diagnosis, comorbid diseases, preoperative physical examination findings and laboratory results, hospital stay after operation, and form of discharge.

Patients who were administered cardiopulmonary resuscitation (CPR) immediately before the operation, those who had CPR on the operation table, and pregnant cases were excluded from the study.

Using the same electronic database and hospital files, Acute Physiology and Chronic Health Evaluation II (APACHE II) score,^6^ Simplified Acute Physiology Score II (SAPS II),^7^ Porthsmouth Physiological and Operative Severity Score for enumeration of mortality and morbidity (P-POSSUM)^8^ Surgical Apgar Score (SAS),^9^ Goldman multifactorial risk index for non-cardiac surgeries (GCRI),^10^ and Charlson Comorbidity Index (CCI)^11^ values of these patients were determined from the preoperative 24-hour data and intraoperative records according to their definitions.

### Statistical Analysis

All analyses were completed using SPSS 15 (SPSS Inc, Chicago, IL) program. Descriptive variables are given as frequency and percentage, whereas continuous variables are given as mean, standard deviation, median and minimum-maximum values. Continuous variables of exitus and surviving patient groups were compared with the nonparametric Mann-Whitney *U* test, whereas the correlation with form of discharge and other descriptive statistics was investigated with the *χ*^2^ test. The investigated risk scores were evaluated for mortality estimation strength with ROC analysis. The statistically significant risk scores for area under the curve in ROC analysis and cutoff values were determined with Youden Index method. The study was completed with 95% confidence interval. Statistical significance was accepted as *P* < 0.05.

## RESULTS

Data of 101 ASA-PS V patients operated in between 2011 and 2013 were retrospectively evaluated. Data from 2 of these patients were not complete, 3 were pregnant undergoing emergency cesarean section, and 7 patients were undertaken to the operation room with CPR and they were all excluded from the study. The remaining 89 patients included in the study were 54 males (60.7%) and 35 females (39.3%). Mean age of males and females was 64.4 ± 18.41 and 65.4 ± 18.91 years, respectively without a statistical significance (Table 1) (*P* > 0.05). Patients’ 24-hour survey and observed mortality, diagnoses, and types of operation are presented in Table 2.

**TABLE 1:**
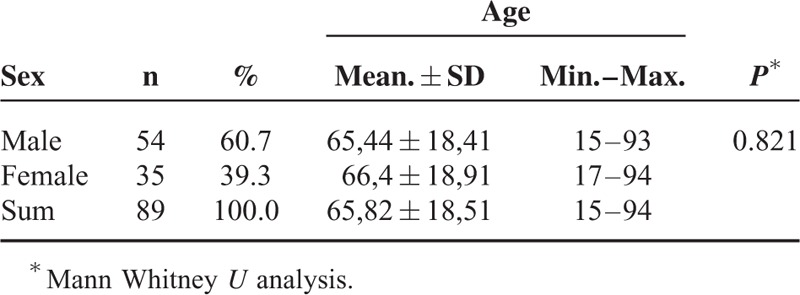
Mean Age Distribution of Cases According to Sex

**TABLE 2:**
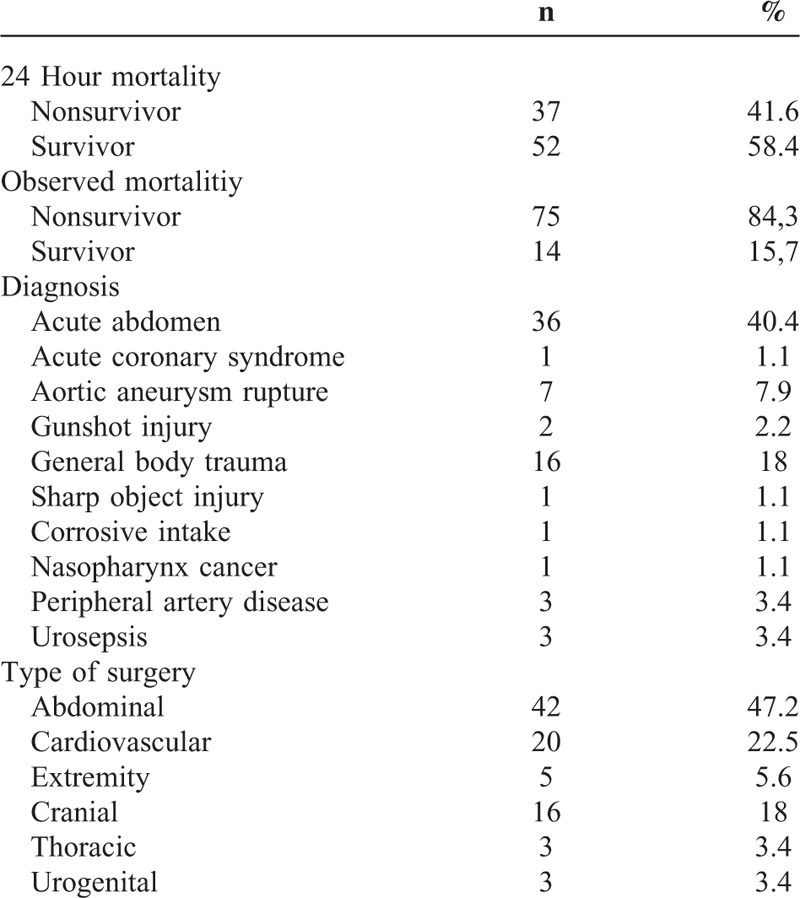
Mortality Rates, Diagnoses, Surgery Types of the Patients

When the mean distribution of hospital stay and APACHE II, P POSSUM, CCI, GCRI, and SAPS II scores are investigated in terms of form of discharge, the hospital stay of exitus patients was found to be statistically significantly low compared with the hospital stay of surviving patients (*P* < 0.05). The APACHE II- and SAPS II-predicted mortality values of exitus patients were found to be statistically significantly high compared with these values in surviving patients (*P* < 0.05). In terms of other variables, there was no statistically significant difference (*P* > 0.05). Data are shown in Table 3.

**TABLE 3:**
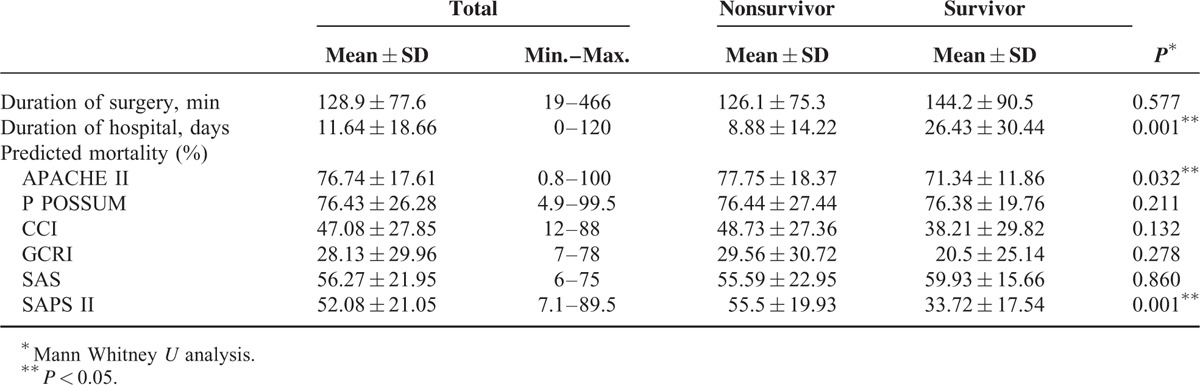
Mean Distribution of Surgical Duration, Hospital Stay, and Predicted Mortality Rates for APACHE II, P POSSUM, CCI, GCRI, SAS, and SAPS II Scores

**FIGURE 1:**
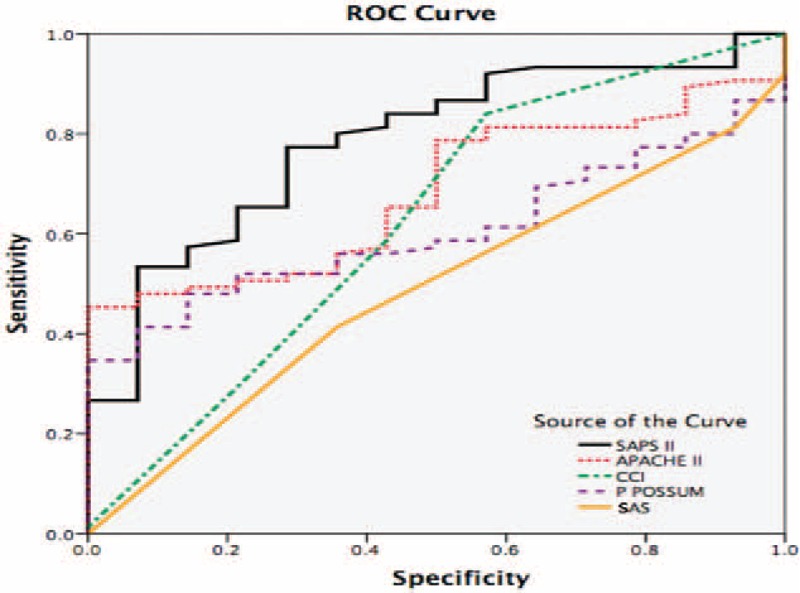
Receiver operator characteristic analysis results for predicted mortality rates of SAPS II, APACHE II, CCI, P POSSUM, and SAS scores.

According to the area under the curve in ROC analysis, predicted mortality rates of APACHE II and SAPS II mortality results were found to be statistically significant for estimation of mortality (*P* < 0.05). P POSSUM, GCRI, CCI, and SAS mortality results were not found to be statistically significant for estimation of mortality (*P* > 0.05) (Table 4, Figure 1).

**TABLE 4:**
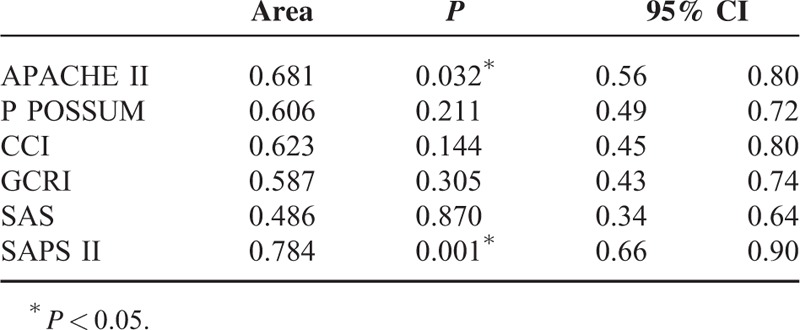
ROC Curve Analysis Results for Predicted Mortality Rate of APACHE II, P POSSUM, CCI, GCRI, SAS, and SAPS II scores

The cut-off for SAPS II score was determined as 41. At this value, the sensitivity was 77.3%, specificity was 71.4%, and accuracy was 76.4%. Of 27 cases with SAPS II score <41, 17 (63%) had exitus and 10 (37%) survived, whereas of 62 cases with SAPS II score >41, 58 (93.5%) had exitus and 4 (6.5%) survived (*P* = 0.001). The cut-off for APACHE II score was determined as 85.8. The sensitivity was 45.3%, specificity was 100% and accuracy was 53.9% at this value. Of 55 cases with APACHE II score <85.8, 41 (74.5%) had exitus and 14 (25.5%) survived, whereas of the 34 cases with SAPS II score >85.8, 34 (100%) had exitus (*P* = 0.001). Data are shown in Table 5.

**TABLE 5:**
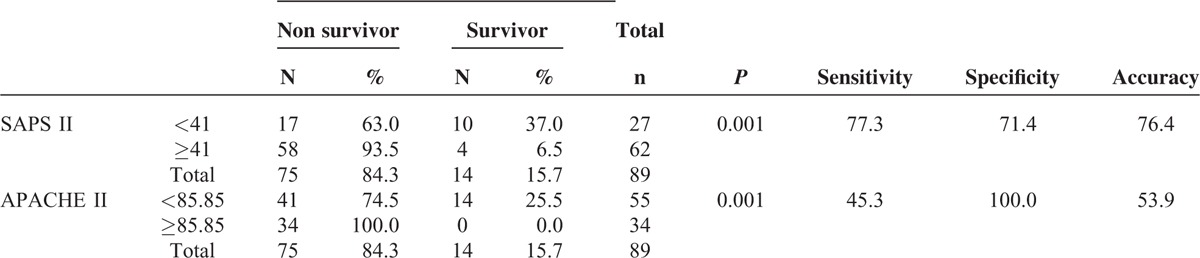
Cut-off Analysis of SASPS II and APACHE II Scores

## DISCUSSION

This research evaluated ASA-PS V, high-risk patients in terms of anesthesia, to compare the mortality estimation of risk evaluation systems and observed the SAPS II and APACHE II mortality estimations calculated in the preoperative period were superior to the other scoring systems. When SAPS II and APACHE II are compared with each other, using logistic regression analysis, the cutoff value of 41 for SAPS II was found to be superior to determine the mortality risk of patients compared with APACHE II.

APACHE II and SAPS II are the most commonly used risk evaluation systems to determine patient mortality especially for intensive care patients. Both risk evaluation systems give points for hematological values, heart and respiratory measurements, kidney functions, Glasgow coma scale, and accompanying chronic diseases to determine a score and estimate short-term mortality. However, as these scores require values from within the first 24 hours, there may be problems in their application to emergency cases.^12^ There are many studies showing SAPS II and APACHE II can be used for risk estimation for general and emergency surgery cases.^13–15^ SAPS II and APACHE II can be affected by treatment during intensive care monitoring, and as a result worst physiological values of patients in the first 24 hours are more valuable for mortality estimation. It was found that SAPS II and APACHE II mortality estimation in intensive care patients may change on different days of treatment. The reason for this is that parameters are affected by the treatment process, may recover with electrolyte treatment, and hypotension may be masked by vasopressor medication.^12^

In a study comparing the mortality rates of 1851 intensive care patients, SAPS III and SAPS II were found to be superior to APACHE II in terms of mortality discrimination.^16^ In an another cohort study comparing intensive care scoring systems in a big database, it was found that APACHE III and SAPS II were superior to APACHE II in terms of distinguishing mortality, which coincides with our research.^17^

Haq et al^18^ in their research on patients older than 90 years who were monitored in intensive care after operations between 2000 and 2010, found the mortality estimations of SAPS III, SAPS II, and APACHE II values. The expected mortality rates according to SAPS II were 57.4 ± 20.0 (55.2% ± 29.7%) for deceased patients and 41.7 ± 14.9 (30.5% ± 23.7%) for surviving patients, similar to the mortality rates in our deceased patients. Additionally, this study found 77% specificity and 65% sensitivity for a cut-off of 44 for SAPS II, very close to our cutoff value of 41.

Since SAPS and APACHE scores did not evaluate data from intraoperative period, another mortality estimation system for surgical cases, POSSUM, has been proposed. POSSUM scoring system evaluates data from 12 physiological parameters and 6 surgical risk parameters to provide morbidity and mortality rates.^19^ Using the same parameters with linear analysis method, Porthsmouth POSSUM (P-POSSUM) scoring system was developed for mortality estimation.^19^ In a study comparing POSSUM with P POSSUM for 145 elective or emergency craniotomy patients, P POSSUM was found to be superior for mortality estimation.^20^ Although P POSSUM was shown to be superior to POSSUM for risk stratification, our analysis demonstrated that APACHE II and SAPS II were superior in ASA PS V subgroup of patients. However, this superiority should be interpreted with a notice to the possibility of positive effects from ongoing therapies within the first 24 hours of patients while data for APACHE and SAPS II came up.

Horwood et al^13^ in research on a limited number and different surgical groups with ASA-PS V found that P POSSUM and APACHE II were superior for mortality estimation compared with ASA. Results of present study are in agreement with Horwood et al's^13^ study in that APACHE II score is discriminative to guess mortality, but in contrary findings are found about P POSSUM. There are certain differences between 2 studies: previous study included only 23 patients, 21 of those patients were classified as ASA-PS V by the same anesthesist; all of the patients were candidates of intra-abdominal surgery and data for POSSUM and APACHE II scores were derived from intraoperative values. In contrast, present study includes almost 4-fold more patients, ASA-PS V classifications were made by different senior anesthesists, and patients underwent different types of surgery. One limitation of P POSSUM score is that it needs data from intraoperative values; thus, it cannot serve as a preoperative predictive tool to estimate mortality, although APACHE II and SAPS II can serve for it. Then, APACHE II, and SAPS II scores can be expected to help clinicians on their decisions for operation, whereas P POSSUM cannot.

In accordance with our findings, in a prospective research of 224 patients with colorectal malignancy, it was found that SAPS II mortality estimation was superior to P POSSUM, POSSUM, and APACHE II.^21^ SAPS II also provided better mortality estimation rates in 48 patients with gas in the portal hepatic vein when compared with SAPS II-, APACHE II-, and sepsis-related organ failure assessment scores.^22^

In a prospective study on 202 intensive care patients, it was found that APACHE II provided better differentiation of short-term hospital mortality compared with APACHE III and SAPS II.^23^ Again another study by O’Dair et al^24^ on 92 patients operated for acute abdomen found POSSUM, APACHE II, and III were superior to SAPS II, different to our research. The different results of APACHE II and SAPS II in this study may be because of not using ASA-PS classification of patients, with different comorbid diseases and lack of standardization of scores calculated in treatment stages. Additionally, both scoring systems do not evaluate perioperative parameters as POSSUM does, which may help to assess different results in the postoperative period.^12^

GCRI provides percentages for cardiac complications that may be observed during noncardiac surgery and CCI gives yearly mortality percentages based on patient's internal diseases.^25^ Studies comparing these risk scoring systems to ASA-PS have shown that ASA-PS classification is superior and concluded both should be evaluated together.^10,26^ These scoring indexes gave inferior estimations of mortality in this ASA-PS V subset of patients in the present study similar to earlier studies indicating the same result with all classes of ASA scoring system.^10,26^

Apart from POSSUM, other risk scoring systems do not evaluate intraoperative factors, but the SAS is a simple scoring system developed to estimate morbidity and mortality after surgery using values from the intraoperative period. Intraoperative heart rate, mean arterial pressure, and estimated blood loss are accepted as important markers of the size of surgery and the patient's reflex response to surgery. SAS varies from 0 to 10 and as the score reduces the mortality rates increase.^27,28^ Retrospective research by Julia et al^29^ on 8501 patients showed that high SAS values in patients undergoing high-risk abdominal surgery were related to postoperative intensive care requirements. However, SAS gave the lowest possibility to accurately estimate the mortality in ASA-PS V subset of patients in the present study.

In our research, all risk classification systems were evaluated together for ASA-PS V patients. Although ASA-PS classification can be criticized as being somehow subjective, recognition of a critically ill patient is not difficult for anesthesists. The exclusion criteria of this study also eliminated patients who were just reanimated, in the hope that patients with very low chance of survival could be eliminated leaving a more uniform risk group. Estimated risks in different surgical groups may not coincide with estimated mortality risk in ASA-PS V-classified patients. For example, predetermined risk expectation already exists for neurosurgery, intra-abdominal surgery candidates.^19,20^ However, the main objective of this study was to find an another risk assumption tool, which would help to validate the estimated risk in all ASA-PS V-classified patients.

There is no single risk classification method for use in the preoperative period to evaluate patient mortality and to aid in surgical decisions for high-risk patients. SAPS II score gives the best match for ASA-PS V-classified patients for estimation of peroperative mortality and when both scales are used together, they will estimate the correct outcome with a 76% accuracy for these patients. The results of this study support addition of SAPS II score for the preoperative evaluation of ASA-PS V-classified patients.

There are certain limitations of the present study. First, this study includes data from a single tertiary care hospital, which could affect patient profile, local availability of facilities for patient care, thus the final outcome. Second, classification of a patient to an ASA-PS V subclass has been performed by different anesthesists of varying seniority, which may produce bias. A multicenter prospective study can overcome these issues.

In conclusion, ASA-PS-V classified patient with a high SAPS II and APACHE II risk score, 41 and 86, respectively, is a poor candidate of survival. SAPS II can be suggested as an additional risk stratification score for predicting mortality in these high-risk patients. However, final decision of operation must be made on clinical basis for each case, as sensitivity rates of risk stratification scores are low.

## References

[1] Cuvillon P Nouvellon E Marret E American Society of Anesthesiologists’ Physical Statussystem: a multicentre Francophone study to analyse reasons for classification disagreement. *Eur J Anaesthesiol* 2011; 28:742–747. 21912242 10.1097/EJA.0b013e328348fc9d

[2] Lupei MI Chipman JG Beilman GJ The association between ASA status and other risk stratification models on postoperative intensive care unit outcomes. *Anesth Analg* 2014; 118:989–994. 24781569 10.1213/ANE.0000000000000187

[3] Sidi A Lobato EB Cohen JA The american society of anesthesiologists’ physical status: category. *V revisited J Clin Anesth* 2000; 12:328–334. 10960208 10.1016/s0952-8180(00)00168-9

[4] Vacanti CJ VanHouten RJ Hill RC A statistical analysis of the relationship of physical status to postoperative mortality in 68,388 cases. *Anesth Analg* 1970; 49:564–566. 5534668

[5] Marx GF Mateo CV Orkin LR Computer analysis of postanesthetic death. *Anesthesiology* 1973; 39:54–58. 4786951 10.1097/00000542-197307000-00010

[6] Knaus WA Draper EA Wagner DP APACHE II: a severity of disease classification system. *Crit Care Med* 1985; 13:818–829. 3928249

[7] Le Gall JR Lemeshow S Saulnier F A new simplified acute physiology score (SAPS II) based on a European/North American multicenter study. *JAMA* 1993; 270:2957–2963. 8254858 10.1001/jama.270.24.2957

[8] Copeland GP Jones D Walters M POSSUM: a scoring system for surgical audit. *Br J Surg* 1991; 78:355–360. 2021856 10.1002/bjs.1800780327

[9] Gawande AA Kwaan MR Regenbogen SE An apgar score for surgery. *J Am Coll Surg* 2007; 204:201–208. 17254923 10.1016/j.jamcollsurg.2006.11.011

[10] Prause G Ratzenhofer-Comenda B Pierer G Can ASA grade or goldman's cardiac risk index predict peri-operative mortality? A study of 16 227 patients. *Anaesthesia* 1997; 52:203–206. 9124658 10.1111/j.1365-2044.1997.074-az0074.x

[11] Whitmore RG Stephen JH Vernick C ASA grade and charlson comorbidity Index of spinal surgery patients: correlation with complications and societal cos. *Spine J* 2014; 14:31–38. 23602377 10.1016/j.spinee.2013.03.011

[12] Goertz O Gharagozlou AF Hirsch T A long-term comparison of a routine laboratory parameter-based severity score with APACHE II and SAPS II. *J Trauma* 2011; 71:1835–1840. 21537210 10.1097/TA.0b013e3182154f0b

[13] Horwood J Ratnam S Maw A Decisions to operate: the ASA grade 5 dilemma. *Ann R Coll Surg Engl* 2011; 93:365–369. 21943459 10.1308/003588411X581367 PMC3365453

[14] Koperna T Semmler D Marian F Risk stratification in emergency surgical patients: is the APACHE II score a reliable marker of physiological impairment? *Arch Surg* 2001; 136:55–59. 11146778 10.1001/archsurg.136.1.55

[15] Garcea G Ganga R Neal CP Preoperative early warning scores can predict in-hospital mortality and critical care admission following emergency surgery. *J Surg Res* 2010; 159:729–734. 19181337 10.1016/j.jss.2008.08.013

[16] Sakr Y Krauss C Amaral AC Comparison of the performance of SAPS II, SAPS 3, APACHE II, and their customized prognostic models in a surgical intensive care unit. *Br J Anaesth* 2008; 101:798–803. 18845649 10.1093/bja/aen291

[17] Livingston BM MacKirdy FN Howie JC Assessment of the performance of five intensive care scoring models within a large scottish database. *Crit Care Med* 2000; 28:1820–1827. 10890627 10.1097/00003246-200006000-00023

[18] Haq A Patil S Parcells AL The simplified acute physiology score III is superior to the simplified acute physiology score II and acute physiology and chronic health evaluation II in predicting surgical and Icu mortality in the “oldest old”. *Curr Gerontol Geriatr Res* 2014; 2014:934852 doi: 10.1155/2014/934852. 24696680 10.1155/2014/934852 PMC3948612

[19] Mohil RS Bhatnagar D Bahadur L POSSUM and P-POSSUM for risk-adjusted audit of patients undergoing emergency laparotomy. *Br J Surg* 2004; 91:500–503. 15048756 10.1002/bjs.4465

[20] Mercer S Guha A Ramesh V The P-POSSUM scoring systems for predicting the mortality of neurosurgical patients undergoing craniotomy: further validation of usefulness and application across healthcare systems. *Indian J Anaesth* 2013; 57:587–591. 24403619 10.4103/0019-5049.123332 PMC3883394

[21] Can MF Yagci G Tufan T Can SAPS II predict operative mortality more accurately than POSSUM and P-POSSUM in patients with colorectal carcinoma undergoing resection? *World J Surg* 2008; 32:589–595. 18204950 10.1007/s00268-007-9321-y

[22] Seak CJ Ng CJ Yen DH Performance assessment of the Simplified Acute Physiology Score II, the Acute Physiology and Chronic Health Evaluation II score, and the Sequential Organ Failure Assessment score in predicting the outcomes of adult patients with hepatic portal venous gas in the ED. *Am J Emerg Med* 2014; 32:1481–1484. 25308825 10.1016/j.ajem.2014.09.011

[23] Gilani MT Razavi M Azad AM A comparison of simplified acute physiology score II,acute physiology and chronic health evaluation II and acute physiology and chronic health evaluation III scoring system in predicting mortality and length of stay at surgical intensive care unit. *Niger Med J* 2014; 55:144–147. 24791049 10.4103/0300-1652.129651 PMC4003718

[24] O’Dair GN Leaper DJ Sequential physiology scoring facilitates objective assessment of resuscitation in patients with an intra-abdominal emergency. *Br J Surg* 2003; 90:1445–1450. 14598430 10.1002/bjs.4299

[25] Pantoja Muñoz HJ Fernández Ramos H Guevara Tovar WL Sensitivity, specificity and predictive values of the goldman, detsky and Lee cardiac indices. *Rev Colomb Anestesiol* 2014; 42:184–191.

[26] Tan WP Talbott VA Leong QQ American society of anesthesiologists class and charlson's comorbidity index as predictors of postoperative colorectal anastomotic leak: a single-institution experience. *J Surg Res* 2013; 184:115–119. 23830360 10.1016/j.jss.2013.05.039

[27] Regenbogen SE Ehrenfeld JM Lipsitz SR Utility of the surgical apgar score: validation in 4119 patients. *Arch Surg* 2009; 144:30–36. 19153322 10.1001/archsurg.2008.504

[28] Haynes AB Regenbogen SE Weiser TG Surgical outcome measurement for a global patient population: validation of the surgical apgar score in 8 countries. *Surgery* 2011; 149:519–524. 21216419 10.1016/j.surg.2010.10.019

[29] Sobol JB Gershengorn HB Wunsch H The surgical apgar score is strongly associated with intensive care unit admission after high-risk intraabdominal surgery. *Anesth Analg* 2013; 117:438–446. 23744956 10.1213/ANE.0b013e31829180b7 PMC4020414

